# Reference Ranges of Glycemic Variability in Infants after Surgery—A Prospective Cohort Study

**DOI:** 10.3390/nu14040740

**Published:** 2022-02-10

**Authors:** Halla Kaminska, Pawel Wieczorek, Grzegorz Zalewski, Beata Malachowska, Przemyslaw Kucharski, Wojciech Fendler, Lukasz Szarpak, Przemyslawa Jarosz-Chobot

**Affiliations:** 1 Department of Children’s Diabetology, School of Medicine in Katowice, Medical University of Silesia, 40-752 Katowice, Poland; przemka1@tlen.pl; 2 Pediatric Intensive Care Unit (PICU), John Paul II Upper Silesian Health Centre in Katowice, 40-752 Katowice, Poland; paulus.vesper@gmail.com; 3 Department of Pediatric Cardiac Surgery, John Paul II Upper Silesian Child Health Center in Katowice, 40-752 Katowice, Poland; grzegorzrafalzalewski@gmail.com; 4 Department of Biostatistics and Translational Medicine, Medical University of Lodz, 90-419 Lodz, Poland; b.e.malachowska@gmail.com (B.M.); kucharskiprzemyslawpiotr@gmail.com (P.K.); fendler.wojciech@gmail.com (W.F.); 5 Department of Radiation Oncology, Albert Einstein College of Medicine, Bronx, NY 10461, USA; 6 Institute of Applied Computer Science, Lodz University of Technology, 90-537 Lodz, Poland; 7 Department of Radiation Oncology, Dana-Farber Cancer Institute, Boston, MA 02155, USA; 8 Henry JN Taub Department of Emergency Medicine, Baylor College of Medicine, Houston, TX 77030, USA; lukasz.szarpak@bcm.edu; 9 Institute of Outcomes Research, Maria Sklodowska-Curie Medical Academy, 03-411 Warsaw, Poland

**Keywords:** glycemic control, heart defects, congenital, intensive care units, pediatric, reference values, postoperative care, thoracic surgery

## Abstract

We aimed to define reference ranges of glycemic variability indices derived from continuous glucose monitoring data for non-diabetic infants during post-operative intensive care treatment after cardiac surgery procedures. We performed a prospective cohort intervention study in a pediatric intensive care unit (PICU). Non-diabetic infants aged 0–12 months after corrective cardiovascular surgery procedures were fitted upon arrival to the PICU with a continuous glucose monitoring system (iPro2, Medtronic, Minneapolis, MN, USA). Thirteen glycemic variability indices were calculated for each patient. Complete recordings of 65 patients were collected on the first postoperative day. During the first three postsurgical days 5%, 24% and 43% of patients experienced at least one hypoglycemia episode, and 40%, 10% and 15%—hyperglycemia episode, respectively, in each day. Due to significant differences between the first postoperative day (mean glycemia 130 ± 31 mg/dL) and the second and third day (105 ± 18 mg/dL, 101 ± 22.2 mg/dL; *p* < 0.0001), we proposed two separate reference ranges—for the acute and steady state patients. Thus, for individual glucose measurements, we proposed a reference range between 85 and 229 mg/dL and 69 and 149 mg/dL. For the mean daily glucose level, ranges between 122 and 137 mg/dL and 95 and 110 mg/dL were proposed. In conclusion, rt-CGM revealed a very high likelihood of hyperglycemia in the first postsurgical day. The widespread use of CGM systems in a pediatric ICU setting should be considered as a safeguard against dysglycemic episodes; however, reference ranges for those patients should be different to those used in diabetes care.

## 1. Introduction

Fluctuations of blood glycemia levels, (dysglycemia) defined as hyper/hypoglycemia, are observed in approximately 90% of patients who are admitted to the intensive care unit (ICU), regardless of previously diagnosed diabetes [1]. Unfortunately, the literature regarding glycemic monitoring in the ICU setting in neonatal children is scarce, and to date, no guidelines have been issued regarding this aspect of diabetes management. With the onset of modern glucose monitoring devices, it is now possible to monitor glycemia in real time with the rt-CGM system (real-time continuous glucose monitoring), which alleviates the decision-making process in diabetes treatment [2]. Additionally, the use of CGM allows for the decrease in the frequency of heel prick blood sampling [3], thus reducing the pain-related stress and its long-term sequelae [4]. CGM opened new possibilities to monitor patients likely to experience rapid changes of blood glucose levels. A potential target group for this intervention are patients treated in intensive care units or undergoing major surgical procedures. In such individuals, a hypermetabolic condition develops, resulting from increased gluconeogenesis, exacerbated by a peripheral insulin resistance along with a defect in pancreatic islet beta cell secretion as a complex interaction between counter-regulatory hormones and inflammatory cytokines [5]. This adds to the myriad of physiologic challenges in the critically ill and is associated with an increased risk of severe complications and mortality [6]. As a countermeasure, current guidelines for treating adult patients in critical conditions recommend monitoring blood glucose levels and treating hyperglycemia, aiming to keep blood glucose levels between 100 and 150 mg/dL (Society of Critical Care Medicine [7] or 140 and 180 mg/dL according to the American Diabetes Association (ADA)). According to the recommendations of Battelino et al. [8] published in 2019, the range of desired glycemic range is considered to be between 70 mg/dL and 180 mg/dL for patients with diabetes. Blood glucose in ICU conditions is monitored by analyzing arterial blood gas (ABG), venous blood, arterial blood or rarely by handheld glucometers. It is important to underline that neither of the aforementioned methods allows for detection of fluctuations of glycemia with an under-detection rate of 4–15% of such episodes when considering the group of patients who are in life threatening conditions [9]. However, regular—even frequent—blood glucose measurements when analyzing ABG still do not diagnose a high percentage of glycemic excursions [10].

Therefore, a unique major asset of CGM is that the technique allows for the evaluation of not only average glycemic levels but also the daily or short-term variability of glycemia. A number of indices were proposed to quantify this aspect of glycemic homeostasis, but even the most basic measures show a strong association with the outcomes of the critically ill [11]. Despite this, very little attention is given to the variability aspect of glucose levels in critical care, particularly pediatric. This remains a major yet surprisingly unexplored area, as higher glycemic variability is an indicator of both higher hyper- and hypoglycemic events which also have a detrimental impact in both the short and long term. The occurrence of hypoglycemia was already shown to be an independent factor influencing mortality in the group of patients suffering from life threatening conditions [12]. It was highlighted that even moderate hypoglycemia defined as blood glucose < 80 mg/dL may have a negative impact on a patient’s treatment process and increase the risk of death in adults. Additionally, severe dysglycemia is a negative prognostic factor when treating patients who are in immediate life threating conditions [13]. Survivors of ICU stays also face delayed sequelae of past glycemic dysregulation as both hypo- and hyperglycemia impact neurodevelopment. Sadly, despite these dangers, the optimal protocol for small children treated in ICUs has not been established, and as such, even the reference ranges of glycemic parameters remain largely arbitrary. Those who are hospitalized in pediatric or neonatal ICUs due to major surgical procedures are thus treated in a reactive manner, which puts them at risk of complications of either the hypermetabolic state, glycemic excursions or adverse effects of overtreatment with insulin or supplemental glucose. To counter this, we designed a cohort study to evaluate the glycemic patterns in infants undergoing cardiac surgeries with the aim to define the reference range of glycemic variability observable in such individuals and to identify factors impacting on impaired glycemic homeostasis.

## 2. Materials and Methods

### 2.1. Participants

The study was designed as a prospective cohort study and was conducted in accordance with the Strengthening the Reporting of Observational Studies in Epidemiology (STROBE) Statement [14]. This study was performed in a single third-level Cardiac Surgery Unit and Pediatric Intensive Care Unit (PICU) John Paul II Upper Silesian Health Centre in Katowice, Poland. The study was approved by the Bioethical Committee of KNW/0022/KB1/154/16/17. Informed consent was obtained from the children’s parents. Recruitment was performed between 7 August 2017 and 25 April 2019. Eligible patients were defined as children under the age of 1 scheduled to undergo cardiovascular surgical procedures. Exclusion criteria were set as follows: previously diagnosed diabetes, previous episodes of hypoglycemia in anamnesis or detected in laboratory studies and the presence of genetic syndromes associated with cardiac malformations and diabetes. Detailed clinical data before and after surgery were collected as well as for at least the first 3 days since surgery during PICU hospitalization by medical professionals, specialized in pediatric intensive care. All laboratory measurements were performed in the in-hospital diagnostic laboratory using standardized methods, unchanged throughout the study period.

To define the age-specific means and standard deviations, we assumed that a group of at least 65 patients with good-quality CGM system (rt-CGM) data would be a sufficiently robust sample size. Less-intensive studies of infant glycemic levels used sample sizes of ~200 children, but due to the intensiveness of CGM and the rarity of patients undergoing cardiovascular surgery procedures, the sample size of 65 children was deemed as reasonable to meet the goals of the study. Using projections from previous years, we assumed that during the study period, it was likely there would be at least 35 eligible children per year and set the recruitment time accordingly.

Parents or legal guardians of eligible patients were asked about their children’s participation, and if consent was given, the patients had the rt-CGM installed by a team member trained in the procedure using the standardized protocol described below. The rt-CGM (iPro2, Medtronic, Minneapolis, USA) was placed on children’s lower abdomen subcutaneously 2 cm laterally from umbilicus just after arriving to the PICU after the surgery was performed (Figure S1). The placement of the rt-CGM in the abdomen rather than in the arm (as recommended by the manufacturer) was dictated by the small size of our patients and the fact that during blood flow centralization, the extremities are under perfused; thus, the rt-CGM readings could be rendered unusable. If the sensor was removed due to local adverse events or technical errors, reinsertion on the opposite site would be attempted, and if this was impossible, the rt-CGM recordings were discontinued. rt-CGM was calibrated against arterial glucose. MARD (mean absolute relative difference) was calculated for patients with at least 4 measurements paired with 10 min and with properly matched time and day of the calibration (Pearson coefficient ≥ 0.7) and equaled 8.94 ± 3.64%. The minimum and maximum MARD were 2.19% and 16.67%, respectively, with the median equaling 8.7%.

### 2.2. rt-CGM Metrics Analysis

rt-CGM data were exported from device reports as glucose concentration and timestamp of the measurement. Data for each patient were processed using a Python script, where GV (glucose variability) indices were calculated separately. GV indices were only calculated on existing data, returning no value where calculation was not possible due to data loss. Missing data imputation techniques were not used. The calculated parameters were: MBG (mean blood glucose), SD (standard deviation), CV (coefficient of variation), median, GMI (Glucose Management Indicator), CONGA (continuous overall net glycemic action) 6 h, GRADE (Glycemic Risk Assessment Diabetes Equation score), LBGI (low blood glucose index), HBGI (high blood glucose index), ADDR (average daily risk range), J, m100, and TIR (time in range), TAR (time above range), TBR (time below range) and hypo- and hyperglycemia episodes for lower thresholds: 54, 70 and upper thresholds 180 and 250 mg/dL (Supplementary Table S2). Parameters for each day were calculated as the 24 h period following the insertion of the sensor. If there were less than or equal to 173 measures per day (<60% of 288 daily measurements), parameters were not calculated for that day. The normoglycemia range was considered to be between 70 mg/dL and 180 mg/dL, as defined by Battelino et al. [8]. Hyperglycemia and hypoglycemia episodes were diagnosed when the episode lasted at least 15 min.

### 2.3. Statistical Analysis

Continuous data were presented as means ± SD. Nominal data were presented as Ns and percentages. A 95%CI (confidence interval) for mean was used to set the reference range for glycemic variability metrics. The 5th and 95th percentile ranges were used for setting reference range for singular blood glucose measurement. All statistical analyses were carried out with the use of STATISTICA 13.1 (TIBCO Software, Palo Alto, CA, USA). Study was conducted in accordance to the Strengthening the Reporting of Observational Studies in Epidemiology (STROBE) guidelines (Table S3). 

## 3. Results

### 3.1. Study Group Characteristics

**Figure 1:**
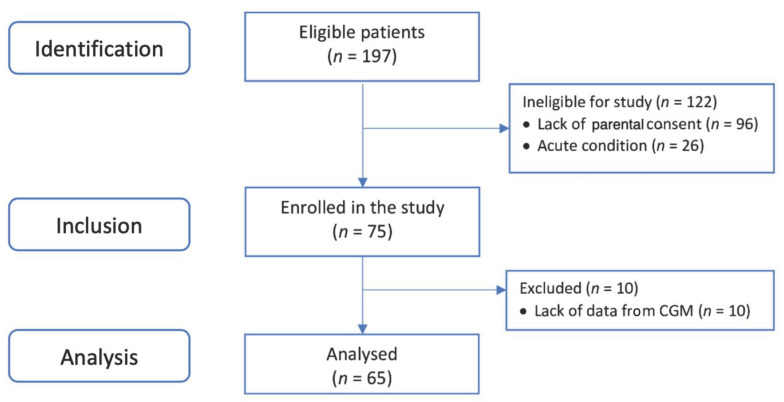
Patients’ selection flowchart.

Among the 197 eligible patients operated on during the study period, rt-CGM sensors were fit in 65. The exclusion of 10 patients during the first 24 h was the result of discharge from the PICU or worsening of the patients’ conditions, which resulted in the need for an operation or resuscitation. The patients’ flow throughout the observation period is shown on Figure 1. rt-CGM metrics were only calculated separately for each day if more than 60% of data were available for this day. This approach resulted in having 65 (100%) eligible patients’ data on day one, 95% on day two, 71% on day three, 49% on day 4, 32% on day 5, 22% on day 6 and 9% on day 7. We focused our analysis on the first 3 post-operative days. Half of the patients were boys (33/65, 50.77%) and the mean children’s age was 4.1 ± 2.9 months (Table 1).

Almost all patients underwent the cardioplegia procedure (91%), with was mostly the crystalloid procedure (66.2%). The most common cardiac defect among the children was common atrioventricular canal (23%) followed by tetralogy of Fallot (15%), hypoplastic left heart syndrome (9%) and isolated ventricular septal defect (8%). During first three post-operative days, most patients were treated with pressor amines (95%, 92.59%; 90%; 85% for each day, respectively), had fever (80%; 38, 61%; 8, 39%) and were under sedation (95%; 92%; 80%).

### 3.2. rt-CGM Metrics

**Figure 2:**
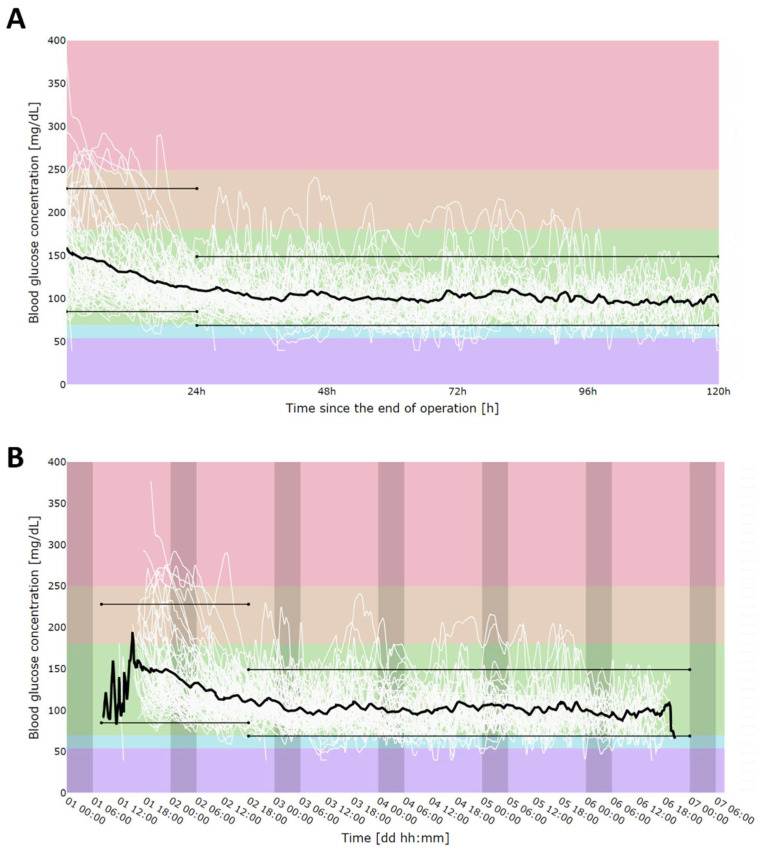
CGM measurement alignment in time since sensor placement (**A**) and in time of day (darker areas show nighttime) (**B**). Solid, black, bold line represents mean. Colors shows different thresholds areas according to current rt-CGM reporting guidelines: hyperglycemia: red (>250 mg/dL), orange (180–250 mg/dL); normoglycemia: green (70–180 mg/dL), hypoglycemia: blue (70–54 mg/dL), violet (>54 mg/dL). Black, thin, parallel lines represent 5th (85 mg/dL for first day and 69 mg/dL for consecutive days) and 95th (228 mg/dL for first day and 149 mg/dL for consecutive days) percentiles for individual rt-CGM measurements. As the first day had significantly higher mean glucose concentration values due to the acute post-operative state, percentiles were calculated separately for the first 24 h after surgery and for the reminder of the post-operative stay (24 h–120 h after the surgery).

Patients’ rt-CGM measurements overlap is shown in Figure 2A, where overlap was perceived based on time since rt-CGM placement (since the surgery) and with the day–night cycle division shown in Figure 2B. We observed that the first 24 h post-operative period was characterized by much higher mean glucose values; we decided to use these data separately as indicators of glucose control in acute post-operative status, and the following days were used as the steady post-operative period. Firstly, we calculated the 5–95th percentile range for individual glucose measurements. For the first 24 h since surgery, this range equaled 85–228 mg/dL, and for the following days, 69–149 mg/dL.

Next, glucose variability (GV) indices were calculated separately for each day. We calculated the means, standard deviations and 95%CI (confidence interval) for all GV indices, primarily for the first 3 days, as likely reference ranges in this group of patients (Table 2). For days 4–5, the data are compiled and placed in Tables S1 and S2, but due to the limited number of individuals, we would advise those reference ranges to be considered more cautiously.

**Figure 3:**
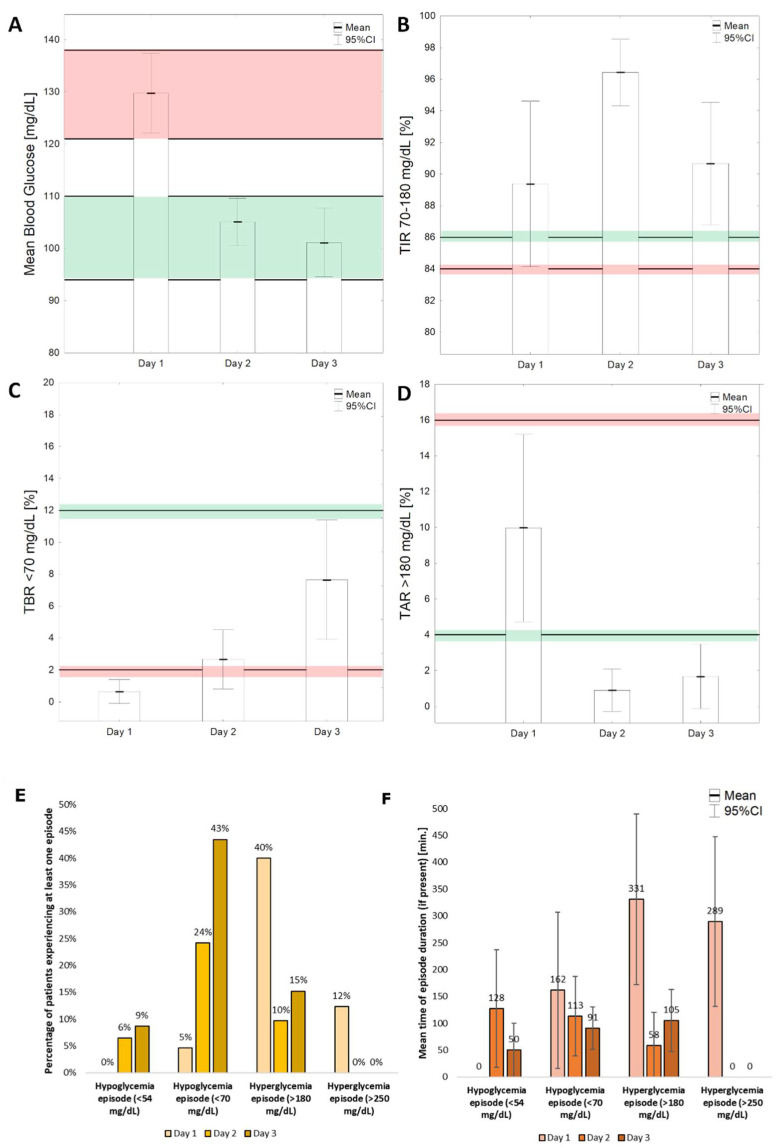
Selected glucose variability indices calculated for first three post-operative days. (**A**)—MBG—mean blood glucose; (**B**)—TIR (time in range) 70–180 mg/dL; (**C**)—TBR (time below range) < 70 mg/dL; (**D**)—time above range > 180 mg/dL. Red parallel lines show reference ranges for the initial 24 h, while green ones are used for subsequent postoperative days. Percentage (**E**) and mean time (**F**) of episodes of hypo- and hyperglycemia during first 3 post-operative days.

The percentage of time in range (TIR) (70–180 mg/dL) increased on the second day but then decreased on the third day. This was mostly due to decrease in the percentage of TAR (time above range) (>180 mg/dL) on the second day and the increase in the percentage of time below range (TBR) (<70 mg) on the third day (Figure 3).

### 3.3. Hypo- and Hyperglycemia Episodes

Three patients (5%) had at least one episode of hypoglycemia on the first post-operative day; however, none of them had glucose levels lower than 54 mg/dL at any time. On the second day, this percentage increased to 24% (25/62) for <70 mg/dL episodes and to 6% (4/62) for <54 mg/dL episodes, and on the third day to 43% (20/46) and 9% (4/46), respectively (Figure 3E).

On the first post-operative day of glucose monitoring, 40% of patients experienced at least one episode of hyperglycemia, and 12% of patients had at least one episode of hyperglycemia above 250 mg/dL. On the second day, the percentage of patients with a hyperglycemia episode above 180 mg/dL dropped to 10%, and none of the patients had an episode of higher hyperglycemia (>250 mg/dL). On the third day, 15% of patients had at least one episode of hyperglycemia above 180 mg/dL (Figure 3E). The mean times of episodes for each consecutive day are shown in Figure 3F.

## 4. Discussion

Our study is the first to present the “normal” range of postoperative glucose variability in a large cohort of infants who underwent cardiovascular surgery. The results show that reactive hyperglycemia during the first postoperative day is commonplace but quickly resolves, although the dynamics of it differ strongly between individuals. Conversely, only a very small percentage of patients develop hypoglycemia, which also resolves spontaneously. While hyperglycemia is a known phenomenon in the postoperative period [14], hypoglycemia, on the other hand, is a far less common finding [15].

Both of these glycemic excursions outside the normal ranges have been known to worsen the prognosis [16] since the inception of modern age medicine; however, it is the combination in the sequence of hyper and hypo glycemic excursions due to the treatment and counter regulatory mechanisms that results in the highest increase in terms of both mortality and morbidity especially infections [17].

Monitoring blood glucose and reductions in glycemic excursions provides patients with the higher chance of a complication-free post-operative period [18,19,20,21,22] and therefore should be a standard of care in the ICU setting [23]. These observations are true for the adult population in critical conditions [24]. However, to date, there are just a handful of studies regarding the observation of blood glucose levels in diabetic [25] and non-diabetic children in the ICU setting. Therefore, our study allows for the assessment of both the clinical utility of rt-CGM in the surgical ICU ward as well as the feasibility of the system.

The important finding was that no device influenced the workflow of the rt-CGM sensor, nor did the sensor disrupt the functionality of the other life-support machines, which is a known problem in critical care medical equipment [26,27]. The CGM system is particularly useful in the detection of clinically silent dysglycemic episodes [28], which was also observed in our study. The utility of rt-CGM in providing a doctor with insight into rapidly changing glucose levels in the critically ill is undeniable [29]. The stress response to surgery incites massive hyperglycemia, in line with prior studies by Cely et al. [30]. This justifies continuous rt-CGM use in this period to allow for rapid interventions and the avoidance of hyperglycemia and hypoglycemia—both factors that were reported to worsen prognoses [31]. However, we must note that during the analyzed period, the time below range increased, indicating the need to adjust insulin dosing so as to not overtreat diabetic patients, as hypoglycemia also negatively affects prognoses [32,33].

Our work has several limitations inherent to its type and studied population. The evaluated group is fairly small, but it is representative given the rarity of patients meeting the inclusion criteria and the high reluctance of parents to participate in any additional research, such as non-therapeutic procedure interventions, in such small children [34].

The extent of surgery may have resulted in varied levels of tissue damage, pain and stress of the patients, but since all children were given adequate analgesic doses, it is unlikely that this factor skewed the results substantially. We aimed to show the reference range of glycemia on standard management in the postoperative period with as few interventions as possible. None of the patients were given insulin with hypoglycemizing agents, and the managing team was blinded towards rt-CGM measurements but had access to laboratory blood glucose concentration measurement results. Patients’ treatment was not modified unless blood glucose levels fell below 54 mg/dL when additional glucose boluses were implemented, potentially altering the low-end glycemic levels. As all patients received multiple drugs and most were given pressor amines and were under sedation, it was impossible to evaluate each drug effect on the glycemia level. We believe that this kind of analysis is out of the scope for this article and might be confusing in its interpretation due to overlapping effects of drugs and their interactions. Finally, we excluded patients with coexisting metabolic conditions and did not evaluate long-term cardiovascular follow-up. Nevertheless, despite these limitations, our work presents the landscape of glycemic variability in infants after major surgeries.

## 5. Conclusions

rt-CGM in the postsurgical period of infants who underwent cardiovascular surgeries reveals a high likelihood of hyperglycemia, potentially exacerbated by clinical factors associated with stress and fluid management. Hypoglycemia is a rare but severe threat that can may occur on later postoperative days and can be reliably identified by CGM use. Therefore, despite the sensor size and physical limitations of the patients, the widespread use of CGM systems in pediatric ICU settings should be considered.

## Figures and Tables

**Table 1:** Participant characteristics.

Clinical Parameters	N (%) or Mean ± SD
	N Total = 65
Gender, male	33 (50.8%)
Cardioplegia type:	
Crystalloid	46 (66.2%)
del Nido	13 (20.0%)
None	6 (9.2%)
Body mass, g	5323.9 ± 1767.0
Height, cm	64.1 ± 8.6
BSA, m^2^	0.30 ± 0.08
Age, days	128.6 ± 89.3
APGAR	8.9 ± 1.5
Gestational age, weeks	38.5 ± 1.7
Birth weight, g	3111.3 ± 563.3
Glucose concentration before surgery, mg/dL	84.8 ± 13.8
Time of surgery, min.	219.6 ± 76.1
Time of aortic cleft, min.	48.5 ± 23.8

Legend: SD = standard deviation.

**Table 2:** Glucose variability (GV) indices for first 3 post-operative days.

GV Indices	Day 1		Day 2		Day 3	
	Mean	95%CI	Mean	95%CI	Mean	95%CI
MBG, mg/dL	129.78	122.15–137.41	105.12	100.66–109.58	101.12	94.54–107.71
Median, mg/dL	129.13	120.88–137.38	103.89	99.32–108.45	99.98	93.48–106.48
SD, mg/dL	23.50	19.73–27.26	14.99	13.19–16.79	15.12	12.59–17.64
CV	17.36	15.19–19.54	14.14	12.67–15.62	14.70	12.57–16.82
GMI	6.41	6.23–6.60	5.82	5.72–5.93	5.73	5.57–5.89
Conga 6 h	23.88	20.70–27.06	19.49	16.53–22.44	19.58	15.72–23.43
ADRR	0.0028	0.0025–0.0032	0.0027	0.0023–0.0032	0.0032	0.0026–0.0038
HBGI	4.25	2.85–5.65	1.13	0.74–1.52	1.31	0.69–1.94
LBGI	0.5980	0.37–0.82	1.36	0.80–1.93	1.97	1.45–2.49
J	25.26	21.39–29.14	14.90	13.41–16.39	14.26	12.12–16.41
GRADE	4.26	3.29–5.23	1.87	1.44–2.31	2.08	1.53–2.62
GRADEhyper	27.14	20.00–34.28	8.22	4.41–12.02	7.99	2.80–13.19
TIR70–180 (%)	89.37	84.13–94.62	96.42	94.31–98.54	90.66	86.80–94.53
TIR70–250 (%)	96.44	94.16–98.71	97.33	95.49–99.17	92.34	88.61–96.08
TAR > 180 (%)	9.97	4.73–15.21	0.90	−0.27–2.08	1.68	−0.12–3.47
TAR > 250 (%)	2.91	0.71–5.11	0.00	NA	0.00	NA
TBR < 54 (%)	0.00	NA	0.62	−0.03–1.27	0.34	−0.02–0.70
TBR < 70 (%)	0.66	−0.09–1.40	2.67	0.83–4.51	7.66	3.92–11.39

Legend: CI—confidence interval, MBG (mean blood glucose), SD (standard deviation), CV (coefficient of variation), median, GMI (Glucose Management Indicator), CONGA (continuous overall net glycemic action) 6 h, GRADE (Glycemic Risk Assessment Diabetes Equation score), GV (glucose variability), LBGI (low blood glucose index), NA (not applicable), HBGI (high blood glucose index), ADDR (average daily risk range), J, m100, and TIR (time in range), TAR (time above range), TBR (time below range).

## Data Availability

Data are available on request from the corresponding author.

## Supplementary Materials

The following supporting information can be downloaded at: https://www.mdpi.com/article/10.3390/nu14040740/s1, Figure S1: CGM system placement on one of the study participants’ abdomen, Table S1: Types of surgeries and the presence of comorbidities in the studied group, Table S2: Continuous glucose monitoring metrics for first 5 post-operative days among infants, Table S3: STROBE Statement—Checklist.
