# Supplementary material for: Reference Ranges of Glycemic Variability in Infants after Surgery—A Prospective Cohort Study
Source: Nutrients. 2022 Feb 10;14(4):740. doi: 10.3390/nu14040740 (PMC8878403; doi:10.3390/nu14040740)
Supplement: Supplementary file 1 [file nutrients-14-00740-s001.zip › nutrients-1551378-supplementary.pdf]

Reference Ranges of Glycemic Variability in Infants After Surgery – a Prospective Cohort Study

Supplementary Digital File

CONTENT

Figure S1 – CGM system placement on one of the study participants abdomen............. 2

Table S1. Types of surgeries and the presence of comorbidities in the studied group. ... 3

Table S2. Continuous glucose monitoring metrics for first 5 post-operative days among infants. 4

Table S3. STROBE Statement—Checklist 10

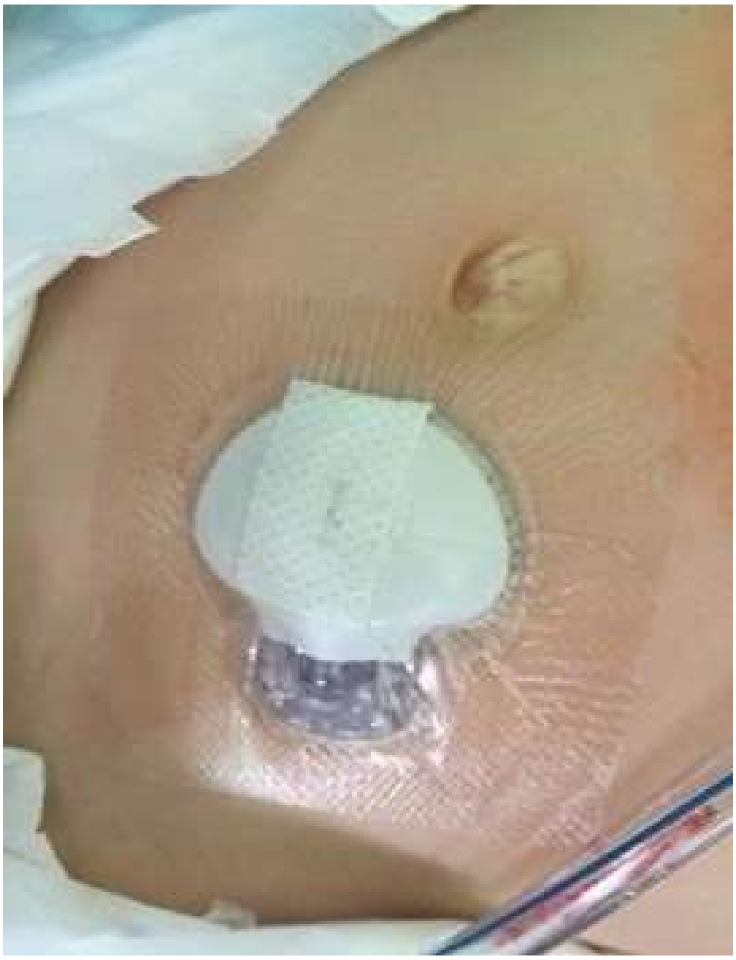

Figure S1 – CGM system placement on one of the study participants abdomen

Table S1. Types of surgeries and the presence of comorbidities in the studied group. Values presented as Ns with %

Type of cardiac defect:	
Complete atrioventricular canal (CAVC)	15 [23.1%]
Tetralogy of Fallot (ToF)	10 [15.4%]
Hypoplastic left heart syndrome (HLHS)	6 [9.2%]
Ventricular septal defect (VSD)	5 [7.7%]
Atrial septal defect (ASD)	3 [4.6%]
Hypoplastic right heart syndrome (HRHS)	3 [4.6%]
Total anomalous pulmonary venous return (TAPVR)	3 [4.6%]
Transposition of the great arteries (TGA)	3 [4.6%]
Double outlet right ventricle (DORV)	3 [4.6%]
Other and complex syndromes	14 [21.5%]
Concomitant diseases:	
None Down	45 [69.2%]
Syndrome DiGeorge+Down	10 [15.4%]
Syndrome Cornelli de Lange	1 [1.5%]
Syndrome	1 [1.5%]
Costello syndrome	1 [1.5%]
Goldenhar syndrome Annular	1 [1.5%]
pancreas Congenital	1 [1.5%]
laryngomalacia Necrotizing	1 [1.5%]
enterocolitis	1 [1.5%]
Solitary kidney	1 [1.5%]
Multiorgan failure	1 [1.5%]

Table S2. Continuous glucose monitoring metrics for first 5 post-operative days among infants. N - number of patients within each day with calculated metric, N% - percentage of patients within each day with calculated metric, -95%CI - lower 95% confidence interval, +95%CI higher 95% confidence interval, SD - standard deviation

	Day 1 (N=65)							
CGM metrics	N	N%	Mean	-95%CI	+95%CI	Minimum	Maximum	SD
ADRR	65	100%	0.0028	0.0025	0.0032	0.0000	0.0061	0.0015
CV	65	100%	17.36	15.19	19.54	7.05	52.93	8.78
Conga6h	65	100%	23.88	20.70	27.06	5.16	68.06	12.82
GMI	65	100%	6.41	6.23	6.60	5.41	8.85	0.74
GRADE	65	100%	4.26	3.29	5.23	0.19	17.67	3.91
GRADE_Hyper	65	100%	27.14	20.00	34.28	0.00	99.65	28.83
HBGI	65	100%	4.25	2.85	5.65	0.01	22.77	5.64
Hypo episodes <54 [N]	65	100%	0.00			0.00	0.00	0.00
Hypo episodes <70 [N]	65	100%	0.06	-0.01	0.14	0.00	2.00	0.30
Hyper episodes >180 [N]	65	100%	0.49	0.33	0.66	0.00	2.00	0.66
Hyper episodes >250 [N]	65	100%	0.17	0.03	0.31	0.00	4.00	0.57
Hypo episodes <54 mean time [min.]	0	0%	0.00	0.00	0.00	0.00	0.00	0.00
Hypo episodes <70 mean time [min.]	3	5%	161.67	16.11	307.22	95.00	205.00	58.59
Hyper episodes >180 mean time [min.]	26	40%	331.06	171.84	490.28	15.00	1255.00	394.20
Hyper episodes >250 mean time [min.]	8	12%	289.38	131.09	447.66	95.00	600.00	189.33
Hyper episodes >180 max time [min.]	65	100%	137.46	63.82	211.11	0.00	1255.00	297.21
Hyper episodes >250 max time [min.]	65	100%	36.08	7.62	64.53	0.00	600.00	114.84
Hypo episodes <54 max time [min.]	65	100%	0.00	0.00	0.00	0.00	0.00	0.00
Hypo episodes <70 max time [min.]	65	100%	8.00	-1.24	17.24	0.00	205.00	37.29
J	65	100%	25.26	21.39	29.14	8.99	74.07	15.64
LBGI	65	100%	0.60	0.37	0.82	0.00	5.04	0.91
MBG [mg/gL]	65	100%	129.78	122.15	137.41	87.99	231.65	30.80
Median [mg/dL]	65	100%	129.13	120.88	137.38	89.00	249.00	33.28
SD [mg/dL]	65	100%	23.50	19.73	27.26	6.84	71.70	15.18

TAR>180 [%]	65	100%	9.97	4.73	15.21	0.00	94.72	21.16
TAR>250 [%]	65	100%	2.91	0.71	5.11	0.00	41.67	8.88
TBR<54 [%]	65	100%	0.00			0.00	0.00	0.00
TBR<70 [%]	65	100%	0.66	-0.09	1.40	0.00	15.23	3.01
TIR54-180 [%]	65	100%	90.03	84.79	95.27	5.28	100.00	21.16
TIR54-250 [%]	65	100%	97.09	94.89	99.29	58.33	100.00	8.88
TIR70-180 [%]	65	100%	89.37	84.13	94.62	5.28	100.00	21.15
TIR70-250 [%]	65	100%	96.44	94.16	98.71	58.33	100.00	9.17
m100	65	100%	115.41	96.72	134.10	24.56	360.00	75.42
	Day 2 (N=62)							
CGM metrics	N	N%	Mean	-95%CI	+95%CI	Minimum	Maximum	SD
ADRR	62	100%	0.0027	0.0023	0.0032	0.0000	0.0065	0.0019
CV	62	100%	14.14	12.67	15.62	5.58	28.41	5.81
Conga6h	62	100%	19.49	16.53	22.44	5.97	65.19	11.65
GMI	62	100%	5.82	5.72	5.93	5.09	7.15	0.42
GRADE	62	100%	1.87	1.44	2.31	0.19	8.16	1.71
GRADE_Hyper	62	100%	8.22	4.41	12.02	0.00	64.88	14.98
HBGI	62	100%	1.13	0.74	1.52	0.00	7.88	1.53
Hypo episodes <54 [N]	62	100%	0.06	0.00	0.13	0.00	1.00	0.25
Hypo episodes <70 [N]	62	100%	0.32	0.16	0.49	0.00	3.00	0.65
Hyper episodes >180 [N]	62	100%	0.13	0.02	0.24	0.00	2.00	0.42
Hyper episodes >250 [N]	62	100%	0.00			0.00	0.00	0.00
Hypo episodes <54 mean time [min.]	4	6%	127.50	17.93	237.07	70.00	225.00	68.86
Hypo episodes <70 mean time [min.]	15	24%	112.78	38.80	186.76	15.00	545.00	133.59
Hyper episodes >180 mean time [min.]	6	10%	58.33	-3.28	119.94	20.00	160.00	58.71
Hyper episodes >250 mean time [min.]	0	0%	0.00	0.00	0.00	0.00	0.00	0.00
Hyper episodes >180 max time [min.]	62	100%	7.42	-1.11	15.95	0.00	215.00	33.61
Hyper episodes >250 max time [min.]	62	100%	0.00	0.00	0.00	0.00	0.00	0.00
Hypo episodes <54 max time [min.]	62	100%	8.23	-0.68	17.13	0.00	225.00	35.08
Hypo episodes <70 max time [min.]	62	100%	31.61	8.17	55.05	0.00	545.00	92.30
J	62	100%	14.90	13.41	16.39	7.72	38.68	5.85

LBGI	62	100%	1.36	0.80	1.93	0.00	15.54	2.23
MBG [mg/gL]	62	100%	105.12	100.66	109.58	74.42	160.72	17.57
Median [mg/dL]	62	100%	103.89	99.32	108.45	74.00	155.00	17.97
SD [mg/dL]	62	100%	14.99	13.19	16.79	5.93	37.13	7.09
TAR>180 [%]	62	100%	0.90	-0.27	2.08	0.00	32.20	4.63
TAR>250 [%]	62	100%	0.00			0.00	0.00	0.00
TBR<54 [%]	62	100%	0.62	-0.03	1.27	0.00	15.63	2.57
TBR<70 [%]	62	100%	2.67	0.83	4.51	0.00	37.85	7.25
TIR54-180 [%]	62	100%	98.48	97.16	99.79	67.80	100.00	5.19
TIR54-250 [%]	62	100%	99.38	98.73	100.03	84.38	100.00	2.57
TIR70-180 [%]	62	100%	96.42	94.31	98.54	62.15	100.00	8.32
TIR70-250 [%]	62	100%	97.33	95.49	99.17	62.15	100.00	7.25
m100	62	100%	74.72	65.50	83.94	23.39	195.45	36.30
	Day 3 (N=46)							
CGM metrics	N	N%	Mean	-95%CI	+95%CI	Minimum	Maximum	SD
ADRR	46	100%	0.0032	0.0026	0.0038	0.0000	0.0067	0.0019
CV	46	100%	14.70	12.57	16.82	3.25	38.93	7.14
Conga6h	46	100%	19.58	15.72	23.43	3.64	68.30	12.99
GMI	46	100%	5.73	5.57	5.89	4.99	7.46	0.53
GRADE	46	100%	2.08	1.53	2.62	0.26	9.83	1.84
GRADE_Hyper	46	100%	7.99	2.80	13.19	0.00	84.75	17.50
HBGI	46	100%	1.31	0.69	1.94	0.00	9.78	2.09
Hypo episodes <54 [N]	46	100%	0.11	0.00	0.22	0.00	2.00	0.38
Hypo episodes <70 [N]	46	100%	1.30	0.71	1.90	0.00	7.00	2.00
Hyper episodes >180 [N]	46	100%	0.20	0.03	0.36	0.00	3.00	0.54
Hyper episodes >250 [N]	46	100%	0.00			0.00	0.00	0.00
Hypo episodes <54 mean time [min.]	4	9%	50.00	0.10	99.90	25.00	90.00	31.36
Hypo episodes <70 mean time [min.]	20	43%	90.97	51.05	130.89	20.00	400.00	85.29
Hyper episodes >180 mean time [min.]	7	15%	104.76	47.06	162.46	35.00	178.33	62.39
Hyper episodes >250 mean time [min.]	0	0%	0.00	0.00	0.00	0.00	0.00	0.00
Hyper episodes >180 max time [min.]	46	100%	19.02	1.64	36.40	0.00	320.00	58.52

Hyper episodes >250 max time [min.]	46	100%	0.00	0.00	0.00	0.00	0.00	0.00
Hypo episodes <54 max time [min.]	46	100%	4.46	-0.46	9.37	0.00	90.00	16.54
Hypo episodes <70 max time [min.]	46	100%	54.89	28.46	81.33	0.00	400.00	89.02
J	46	100%	14.26	12.12	16.41	5.82	42.17	7.22
LBGI	46	100%	1.97	1.45	2.49	0.00	5.66	1.76
MBG [mg/gL]	46	100%	101.12	94.54	107.71	70.06	173.63	22.17
Median [mg/dL]	46	100%	99.98	93.48	106.48	70.00	164.00	21.89
SD [mg/dL]	46	100%	15.12	12.59	17.64	2.51	40.78	8.50
TAR>180 [%]	46	100%	1.68	-0.12	3.47	0.00	37.94	6.04
TAR>250 [%]	46	100%	0.00			0.00	0.00	0.00
TBR<54 [%]	46	100%	0.34	-0.02	0.70	0.00	6.25	1.21
TBR<70 [%]	46	100%	7.66	3.92	11.39	0.00	46.53	12.58
TIR54-180 [%]	46	100%	97.98	96.18	99.79	62.06	100.00	6.07
TIR54-250 [%]	46	100%	99.66	99.30	100.02	93.75	100.00	1.21
TIR70-180 [%]	46	100%	90.66	86.80	94.53	53.47	100.00	13.03
TIR70-250 [%]	46	100%	92.34	88.61	96.08	53.47	100.00	12.58
m100	46	100%	91.76	79.18	104.33	21.08	232.68	42.35
	Day 4 (N=32)							
CGM metrics	N	N%	Mean	-95%CI	+95%CI	Minimum	Maximum	SD
ADRR	32	100%	0.0028	0.0020	0.0035	0.0000	0.0066	0.0020
CV	32	100%	14.59	12.60	16.58	5.57	26.39	5.51
Conga6h	32	100%	20.27	15.98	24.56	7.82	70.20	11.90
GMI	32	100%	5.80	5.61	6.00	4.93	7.61	0.54
GRADE	32	100%	2.16	1.37	2.96	0.11	10.78	2.21
GRADE_Hyper	32	100%	8.87	1.67	16.07	0.00	93.40	19.97
HBGI	32	100%	1.33	0.52	2.14	0.00	10.89	2.25
Hypo episodes <54 [N]	32	100%	0.22	0.04	0.40	0.00	2.00	0.49
Hypo episodes <70 [N]	32	100%	1.03	0.57	1.49	0.00	5.00	1.28
Hyper episodes >180 [N]	32	100%	0.28	-0.03	0.59	0.00	4.00	0.85
Hyper episodes >250 [N]	32	100%	0.00			0.00	0.00	0.00
Hypo episodes <54 mean time [min.]	6	19%	39.17	14.02	64.31	15.00	75.00	23.96

Hypo episodes <70 mean time [min.]	18	56%	64.33	36.06	92.61	15.00	255.00	56.86
Hyper episodes >180 mean time [min.]	4	13%	128.75	0.36	257.14	20.00	192.50	80.69
Hyper episodes >250 mean time [min.]	0	0%	0.00	0.00	0.00	0.00	0.00	0.00
Hyper episodes >180 max time [min.]	32	100%	24.53	-4.25	53.31	0.00	350.00	79.83
Hyper episodes >250 max time [min.]	32	100%	0.00	0.00	0.00	0.00	0.00	0.00
Hypo episodes <54 max time [min.]	32	100%	7.34	0.76	13.93	0.00	75.00	18.27
Hypo episodes <70 max time [min.]	32	100%	57.81	12.38	103.24	0.00	690.00	126.01
J	32	100%	15.12	12.29	17.95	5.96	41.69	7.85
LBGI	32	100%	1.82	1.20	2.44	0.00	7.20	1.72
MBG [mg/gL]	32	100%	104.24	96.04	112.44	67.76	179.93	22.74
Median [mg/dL]	32	100%	104.33	96.02	112.64	68.00	182.00	23.06
SD [mg/dL]	32	100%	15.51	12.72	18.30	5.07	39.14	7.73
TAR>180 [%]	32	100%	3.03	-0.80	6.86	0.00	53.47	10.63
TAR>250 [%]	32	100%	0.00			0.00	0.00	0.00
TBR<54 [%]	32	100%	0.62	0.13	1.11	0.00	5.21	1.36
TBR<70 [%]	32	100%	5.86	1.80	9.92	0.00	53.13	11.27
TIR54-180 [%]	32	100%	96.36	92.56	100.15	46.53	100.00	10.53
TIR54-250 [%]	32	100%	99.38	98.89	99.87	94.79	100.00	1.36
TIR70-180 [%]	32	100%	91.11	85.93	96.30	46.53	100.00	14.38
TIR70-250 [%]	32	100%	94.14	90.08	98.20	46.88	100.00	11.27
m100	32	100%	85.88	69.00	102.76	28.41	250.96	46.81
	Day 5 (N=21)							
CGM metrics	N	N%	Mean	-95%CI	+95%CI	Minimum	Maximum	SD
ADRR	21	100%	0.0025	0.0016	0.0034	0.0000	0.0061	0.0019
CV	21	100%	14.48	12.10	16.86	6.75	25.40	5.23
Conga6h	21	100%	19.06	14.63	23.49	5.15	41.31	9.73
GMI	21	100%	5.64	5.46	5.83	4.93	6.26	0.41
GRADE	21	100%	1.77	1.24	2.30	0.24	4.77	1.16
GRADE_Hyper	21	100%	4.14	0.92	7.37	0.00	21.88	7.09
HBGI	21	100%	0.71	0.29	1.14	0.00	3.28	0.93
Hypo episodes <54 [N]	21	100%	0.24	-0.01	0.48	0.00	2.00	0.54

Hypo episodes <70 [N]	21	100%	1.62	0.64	2.60	0.00	7.00	2.16
Hyper episodes >180 [N]	21	100%	0.10	-0.04	0.23	0.00	1.00	0.30
Hyper episodes >250 [N]	21	100%	0.00			0.00	0.00	0.00
Hypo episodes <54 mean time [min.]	4	19%	32.50	1.69	63.31	15.00	60.00	19.36
Hypo episodes <70 mean time [min.]	14	67%	60.79	28.50	93.09	15.00	227.50	55.93
Hyper episodes >180 mean time [min.]	2	10%	92.50	-256.92	441.92	65.00	120.00	38.89
Hyper episodes >250 mean time [min.]	0	0%	0.00	0.00	0.00	0.00	0.00	0.00
Hyper episodes >180 max time [min.]	21	100%	8.81	-4.46	22.08	0.00	120.00	29.15
Hyper episodes >250 max time [min.]	21	100%	0.00	0.00	0.00	0.00	0.00	0.00
Hypo episodes <54 max time [min.]	21	100%	7.62	-1.84	17.08	0.00	90.00	20.77
Hypo episodes <70 max time [min.]	21	100%	64.52	18.79	110.26	0.00	390.00	100.47
J	21	100%	13.01	10.70	15.32	6.27	23.38	5.08
LBGI	21	100%	2.10	1.17	3.03	0.02	7.62	2.04
MBG [mg/gL]	21	100%	97.57	89.72	105.41	67.90	123.23	17.24
Median [mg/dL]	21	100%	96.95	89.40	104.50	69.00	124.00	16.58
SD [mg/dL]	21	100%	14.42	11.32	17.51	5.01	30.97	6.80
TAR>180 [%]	21	100%	0.63	-0.29	1.56	0.00	8.33	2.03
TAR>250 [%]	21	100%	0.00			0.00	0.00	0.00
TBR<54 [%]	21	100%	0.90	-0.48	2.27	0.00	13.79	3.02
TBR<70 [%]	21	100%	9.49	2.03	16.94	0.00	52.30	16.38
TIR54-180 [%]	21	100%	98.47	96.89	100.05	86.21	100.00	3.47
TIR54-250 [%]	21	100%	99.10	97.73	100.48	86.21	100.00	3.02
TIR70-180 [%]	21	100%	89.88	82.52	97.24	47.70	100.00	16.17
TIR70-250 [%]	21	100%	90.51	83.06	97.97	47.70	100.00	16.38
m100	21	100%	82.03	65.51	98.54	42.69	174.64	36.28

Table S3. STROBE Statement—Checklist

	Item No	Recommendation	Pages
**Title and abstract**	1	(*a*) Indicate the study's design with a commonly used term in the title or the abstract	1
		(*b*) Provide in the abstract an informative and balanced summary of what was done and what was found	1
**Introduction**			
Background/rationale	2	Explain the scientific background and rationale for the investigation being reported	1,2
Objectives	3	State specific objectives, including any prespecified hypotheses	2
**Methods**			
Study design	4	Present key elements of study design early in the paper	3
Setting	5	Describe the setting, locations, and relevant dates, including periods of recruitment, exposure, follow-up, and data collection	3
Participants	6	(*a*) Give the eligibility criteria, and the sources and methods of selection of participants. Describe methods of follow-up	3
		(*b*) For matched studies, give matching criteria and number of exposed and unexposed	-
Variables	7	Clearly define all outcomes, exposures, predictors, potential confounders, and effect modifiers. Give diagnostic criteria, if applicable	3,4
Data sources/ measurement	8*	For each variable of interest, give sources of data and details of methods of assessment (measurement). Describe comparability of assessment methods if there is more than one group	*3,4*
Bias	9	Describe any efforts to address potential sources of bias	4
Study size	10	Explain how the study size was arrived at	-
Quantitative variables	11	Explain how quantitative variables were handled in the analyses. If applicable, describe which groupings were chosen and why	4
Statistical methods	12	(*a*) Describe all statistical methods, including those used to control for confounding	4
		(*b*) Describe any methods used to examine subgroups and interactions	4
		(*c*) Explain how missing data were addressed	4
		(*d*) If applicable, explain how loss to follow-up was addressed	4
		(*e*) Describe any sensitivity analyses	4
**Results**			
Participants	13*	(a) Report numbers of individuals at each stage of study—eg numbers potentially eligible, examined for eligibility, confirmed eligible, included in the study, completing follow-up, and analysed	4
		(b) Give reasons for non-participation at each stage	4
		(c) Consider use of a flow diagram	4

Descriptive data	14*	(a) Give characteristics of study participants (eg demographic, clinical, social) and information on exposures and potential confounders	5
		(b) Indicate number of participants with missing data for each variable of interest	4
		(c) Summarise follow-up time (eg, average and total amount)	4
Outcome data	15*	Report numbers of outcome events or summary measures over time	5,6
Main results	16	(*a*) Give unadjusted estimates and, if applicable, confounder-adjusted estimates and their precision (eg, 95% confidence interval). Make clear which confounders were adjusted for and why they were included	5,6
		(*b*) Report category boundaries when continuous variables were categorized	5,6
		(*c*) If relevant, consider translating estimates of relative risk into absolute risk for a meaningful time period	-
Other analyses	17	Report other analyses done—eg analyses of subgroups and interactions, and sensitivity analyses	-
**Discussion**			
Key results	18	Summarise key results with reference to study objectives	8,9
Limitations	19	Discuss limitations of the study, taking into account sources of potential bias or imprecision. Discuss both direction and magnitude of any potential bias	9
Interpretation	20	Give a cautious overall interpretation of results considering objectives, limitations, multiplicity of analyses, results from similar studies, and other relevant evidence	8,9
Generalisability	21	Discuss the generalisability (external validity) of the study results	9
**Other information**			
Funding	22	Give the source of funding and the role of the funders for the present study and, if applicable, for the original study on which the present article is based	10

*Give information separately for exposed and unexposed groups.

Note: An Explanation and Elaboration article discusses each checklist item and gives methodological background and published examples of transparent reporting. The STROBE checklist is best used in conjunction with this article (freely available on the Web sites of PLoS Medicine at http://www.plosmedicine.org/, Annals of Internal Medicine at http://www.annals.org/, and Epidemiology at http://www.epidem.com/). Information on the STROBE Initiative is available at http://www.strobe-statement.org.
